# Functional Characterization of the VWF p.Cys2163Tyr Variant Reveals Impaired Secretion and Intracellular Processing

**DOI:** 10.3390/biom16081088

**Published:** 2026-07-25

**Authors:** Yuxin Zhang, Yingkun Zhang, Aizhen Yang, Yabei Zuo, Xiaofeng Yan, Feifei Zhang, Yan Wang, Zhiyun Niu, Fengwu Chen, Yi Wu, Jingyu Zhang

**Affiliations:** 1 Department of Hematology, Key Laboratory of Hematology of Hebei Province, The Second Hospital of Hebei Medical University, Shijiazhuang 050000, China; 2 National Clinical Research Center for Hematologic Diseases, Cyrus Tang Medical Institute, Collaborative Innovation Center of Hematology, State Key Laboratory of Radiation Medicine and Prevention, Soochow University, Suzhou 215123, China; 3 Special Examination Department, The Second Hospital of Hebei Medical University, Shijiazhuang 050000, China; 4 The State Key Laboratory of Membrane Biology, School of Life Sciences, Tsinghua University, Beijing 100084, China

**Keywords:** von Willebrand factor, VWF D4 domain, VWD, multimerization, secretion, missense variant

## Abstract

Von Willebrand disease (VWD) is the most common inherited bleeding disorder, yet the contribution of specific VWF domains to its pathogenesis remains incompletely understood. In particular, the role of the D4 domain in VWF secretion, intracellular maturation, and multimer formation has not been fully elucidated. Here, we investigated the functional impact of a heterozygous p.Cys2163Tyr variant located in the D4 domain, identified in a patient with a severe bleeding phenotype, using clinical evaluation, genetic analysis, family studies, and in vitro expression assays. Laboratory testing revealed markedly reduced VWF:Ag (6.8 IU/dL), VWF:GPIbR (0.1 IU/dL), and FVIII:C levels, indicating a severe VWD phenotype in the proband. Plasma VWF multimer analysis showed a markedly reduced overall VWF signal with an almost complete absence of high-molecular-weight multimers, supporting classification of the phenotype as severe type 2A VWD. The heterozygous c.6488G>A (p.Cys2163Tyr) variant was also present in asymptomatic family members, indicating incomplete segregation with the severe phenotype and suggesting that this variant alone is insufficient to explain the proband’s disease severity. Notably, the proband’s mother exhibited mildly reduced VWF levels in the absence of this variant, suggesting the possible contribution of an additional unidentified defect or modifier affecting the maternal allele. In vitro expression demonstrated preserved intracellular VWF antigen, markedly reduced secretion of mutant VWF, and loss of high-molecular-weight VWF multimers. Together, these findings indicate that VWF p.Cys2163Tyr is a functionally deleterious variant that markedly impairs VWF secretion and high-molecular-weight multimer formation in vitro. However, the incomplete segregation observed in the family suggests that this heterozygous variant alone may not fully account for the proband’s severe type 2A VWD phenotype.

## 1. Introduction

Von Willebrand factor (VWF) is a large multimeric plasma glycoprotein that plays a central role in hemostasis. It initiates primary hemostasis by binding to subendothelial collagen at sites of vascular injury and recruiting platelets to form the initial hemostatic plug [1]. In addition, VWF serves as a carrier protein for coagulation factor VIII (FVIII), protecting it from premature clearance and ensuring its availability for secondary hemostasis [2]. The structural integrity and proper function of VWF are essential for maintaining hemostatic balance and play a crucial role in physiological hemostasis.

The VWF precursor consists of a signal peptide, a propeptide (D1–D2), and the mature subunit arranged as D′–D3–A1–A2–A3–D4–B–C1–C2–C3–C4–C5–C6–CK [1,3]. The functional roles of distinct domains have been progressively elucidated, including binding sites for FVIII (D′–D3 domains), platelet glycoprotein Ibα and type VI collagen (A1 domain), types I and III collagen (A3 domain), and platelet GPIIb/IIIa (C4 domain) [1,4]. The A2 domain of VWF contains a cleavage site for the metalloprotease ADAMTS13, which is critical for regulating multimer size [5,6]. The C-terminal cystine knot (CK) domain contains numerous cysteine residues that form both intrachain and interchain disulfide bonds, and the four intrachain disulfide bonds within the CK domain are essential for VWF dimerization and secretion [7].

The functional role of the D4 domain remains incompletely understood. First, the D4 domain contains a binding site for ADAMTS-13 [8,9,10]. Second, through homophilic interactions, D4 mediates strong intermonomer interactions between two VWF monomers, enabling tight side-by-side alignment to form stem structure. In the presence of divalent cations (Ca^2+^, Mg^2+^), this interaction controls the equilibrium between folded and extended dimer conformations, thereby regulating the threshold of VWF’s response to mechanical forces in circulation and maintaining the balance between hemostasis and thrombosis [11]. In contrast, the role of the D4 domain in VWF biosynthesis, secretion, and storage remains elusive. D4 domain mutations have been associated with various types of VWD, and a few variants have been suggested to cause VWF secretion defects [12,13]; however, few of these have been expressed and adequately characterized in vitro.

In this study, we investigated the functional impact of the p.Cys2163Tyr variant located in the VWF D4 domain in a patient with a severe VWD phenotype showing loss of high-molecular-weight multimers. We performed in vitro expression studies in HEK293T cells to evaluate the effects of this variant on VWF expression, secretion, and multimer formation. Our findings provide functional evidence that the p.Cys2163Tyr variant impairs VWF secretion and multimer formation in vitro, supporting a role for the D4 domain in VWF biosynthesis while highlighting the need for cautious interpretation of genotype–phenotype correlation in this family.

## 2. Materials and Methods

### 2.1. Blood Samples and Laboratory Tests

This study was approved by the Medical Ethics Committee of the Second Hospital of Hebei Medical University, and informed consent was obtained from all participants. The proband was a male, aged 5 years and 11 months, of Chinese ethnicity, who presented with epistaxis and gingival bleeding since early childhood. No history of joint bleeding, deep muscle hematoma, or major surgical bleeding was reported. The parents were non-consanguineous. The father and sister denied spontaneous mucocutaneous bleeding, excessive bleeding after minor trauma, or surgery-related bleeding. The mother also denied recurrent epistaxis, gingival bleeding, menorrhagia, postpartum hemorrhage, or excessive bleeding after dental extraction or surgery.

Venous blood was collected using 3.2% sodium citrate as anticoagulant (1:9 ratio), centrifuged at 2500× *g* for 10 min at room temperature, and plasma was collected. Within 2 h of sample collection, activated partial thromboplastin time (APTT), FVIII coagulant activity (FVIII:C), VWF antigen (VWF:Ag), and von Willebrand factor glycoprotein Ib ristocetin-dependent activity (VWF:GPIbR) were measured on an automated coagulation analyzer (ACL TOP 700, Instrumentation Laboratory Company, Bedford, MA, USA) using the manufacturer’s reagents according to the manufacturer’s protocols. Results originally reported as percentages were converted to IU/dL assuming 100% corresponds to 100 IU/dL, according to local laboratory reference standards.

### 2.2. Mutation Screening

Genomic DNA was extracted from peripheral blood of the proband and subjected to targeted next-generation sequencing using a panel covering genes associated with hereditary bleeding and thrombotic disorders (Beijing Mygenostics Co., Ltd., Beijing, China). Sequencing was performed on an Illumina high-throughput sequencing platform, achieving a mean depth of 453× over target regions, with 99.3% and 98.4% of target bases covered at ≥10× and ≥20×, respectively. Variants were called against the GRCh37/hg19 reference genome and annotated using the VWF transcript NM_000552. Variant classification was performed according to the American College of Medical Genetics and Genomics and Association for Molecular Pathology (ACMG/AMP) guidelines [14], and in silico functional predictions were obtained using REVEL, SIFT, PolyPhen-2, MutationTaster, and GERP+.

A candidate variant identified by next-generation sequencing was confirmed by Sanger sequencing in the proband and his available family members (father, mother, and sister). The genomic region containing exon 37 of the VWF gene, where the c.6488G>A (p.Cys2163Tyr) variant is located, was amplified by PCR using specific primers flanking exon 37. The resulting products were purified and subjected to bidirectional Sanger sequencing. Chromatograms were analyzed using SnapGene software (SnapGene 6.0.2, Dotmatics, Boston, MA, USA) to confirm the presence and zygosity of the variant.

### 2.3. Plasmid Construction

The expression vector pSVHVWF containing full-length wild-type human VWF cDNA was generously provided by Professor Evan Sadler (Washington University School of Medicine, St. Louis, MO, USA) [15]. The c.6488G>A (p.Cys2163Tyr) variant was introduced by site-directed mutagenesis using the Q5^®^ Site-Directed Mutagenesis Kit (New England Biolabs, Ipswich, MA, USA) according to the manufacturer’s instructions. Mutagenic primers were designed and synthesized by GENEWIZ (Suzhou, China); primer sequences are available from the corresponding author upon reasonable request. Briefly, PCR amplification was performed using the wild-type pSVHVWF as template, followed by KLD (kinase, ligase, and DpnI) treatment to remove the parental plasmid and circularize the mutated product. The reaction mixture was transformed into *Escherichia coli* DH5α competent cells, and plasmids were purified using a standard mini-prep procedure. Successful introduction of the c.6488G>A mutation was confirmed by Sanger sequencing of the region flanking the mutation site.

### 2.4. Cell Culture and Transfection

Human embryonic kidney 293T (HEK293T) cells were obtained from the Cell Bank of the Chinese Academy of Sciences (Shanghai, China) and cultured in high-glucose Dulbecco’s Modified Eagle’s Medium (DMEM) supplemented with 10% fetal bovine serum (HyClone, Cytiva, Logan, UT, USA) and 1% penicillin–streptomycin, at 37 °C in a humidified atmosphere containing 5% CO_2_.

For transfection, HEK293T cells were seeded in 6-well plates and allowed to reach approximately 70% confluence, then transfected with one of the following plasmid combinations using Lipo8000™ transfection reagent (Vazyme Biotech, Nanjing, China) according to the manufacturer’s instructions: 2.5 μg empty vector, 2.5 μg wild-type VWF (WT), 2.5 μg VWF-p.Cys2163Tyr, or 1.25 μg WT plus 1.25 μg VWF-p.Cys2163Tyr (1:1 ratio, 2.5 μg total) per well. At 72 h post-transfection, conditioned media were collected and clarified by low-speed centrifugation to remove cellular debris. Cells were washed twice with ice-cold phosphate-buffered saline (PBS) and lysed in ice-cold lysis buffer (50 mM Tris-HCl pH 7.4, 150 mM NaCl, 1% Triton X-100) supplemented with 1× cOmplete™ Protease Inhibitor Cocktail (Roche, Basel, Switzerland) and 1 mM phenylmethylsulfonyl fluoride (PMSF). Lysates were cleared by centrifugation at 10,000× *g* for 10 min at 4 °C, and supernatants were collected for subsequent Western blot and ELISA analyses.

### 2.5. Recombinant VWF Protein Expression in Cell Culture

Conditioned media were concentrated 10-fold using 10 kDa molecular weight cut-off (MWCO) centrifugal filter units (Amicon Ultra; Merck Millipore, Burlington, MA, USA). Cell lysate protein concentrations were determined by BCA assay, and equal amounts of total protein (100 μg) were loaded for each sample in a final volume of 20 μL. Samples were mixed with 2× Laemmli Sample Buffer (Bio-Rad, Hercules, CA, USA) containing β-mercaptoethanol (reducing conditions) or without β-mercaptoethanol (non-reducing conditions), and denatured at 100 °C for 10 min before loading.

Proteins were separated on 8% SDS-polyacrylamide gels and transferred onto polyvinylidene difluoride (PVDF) membranes (Merck Millipore, Burlington, MA, USA) by wet electrophoretic transfer at 100 V for 100 min. Membranes were blocked with 5% non-fat dry milk in Tris-buffered saline containing 0.1% Tween 20 (TBST) for 1 h at room temperature, then incubated overnight at 4 °C with a polyclonal rabbit anti-human VWF primary antibody (1:1000; A0082, Dako, Agilent Technologies, Santa Clara, CA, USA) diluted in Primary Antibody Dilution Buffer (Beyotime, Shanghai, China). Membranes were washed three times with TBST (10 min each) and incubated with IRDye^®^ 800CW Goat anti-Rabbit IgG secondary antibody (1:10,000; LI-COR Biosciences, Lincoln, NE, USA) for 1 h at room temperature. After three additional 10 min washes with TBST, fluorescent signals were detected using an Odyssey CLx imaging system (LI-COR Biosciences, Lincoln, NE, USA).

### 2.6. VWF Multimer Analysis

VWF multimer analysis was performed using SDS–agarose gel electrophoresis under non-reducing conditions, as previously described [16]. Briefly, plasma samples or recombinant VWF from cell culture supernatants were separated on 1.5% agarose gels containing SDS and subsequently transferred to membranes for immunoblot detection. Multimers were visualized using a primary antibody against VWF followed by appropriate secondary antibodies. Normal pooled plasma (NHP) was included as a reference control for multimer distribution. The relative distribution of low-, intermediate-, and high-molecular-weight multimers was assessed qualitatively.

### 2.7. Elisa Quantification of VWF Antigen

VWF antigen levels in conditioned media and cell lysates were quantified using a Human von Willebrand Factor (vWF) ELISA Kit (catalog no. JL13750; JONLNBIO, Shanghai, China) according to the manufacturer’s instructions. This kit is a sandwich ELISA with a detection range of 0.78–50 ng/mL and a sensitivity of 0.35 ng/mL. Conditioned media and cell lysates were diluted 1:100 in the universal diluent provided with the kit before assay. Diluted samples (100 μL per well) and serial dilutions of the kit-provided standard (50, 25, 12.5, 6.25, 3.12, 1.56, 0.78, and 0 ng/mL) were added in duplicate to the pre-coated 96-well plate and incubated at 37 °C for 60 min, followed by sequential incubation with biotinylated detection antibody, streptavidin–HRP conjugate, and TMB substrate as described in the manufacturer’s protocol. The reaction was stopped with stop solution, and absorbance was measured at 450 nm on a microplate reader. VWF concentrations (ng/mL) were calculated from the standard curve using four-parameter logistic (4PL) regression and multiplied by the dilution factor. Conditioned media and the corresponding cell lysates from five independent transfection experiments were collected and assayed in a single ELISA run.

### 2.8. Statistical Analysis

Quantitative data are presented as mean ± SD from independent experiments. Normality was assessed using the Shapiro–Wilk test before applying parametric tests. For comparisons between two groups, an unpaired two-tailed Student’s *t*-test was used. Exact *p* values are reported whenever possible, and *p* < 0.05 was considered statistically significant.

## 3. Results

### 3.1. Clinical and Laboratory Phenotype Assessment in the Proband and Family Members

The proband’s complete blood count was unremarkable. Coagulation testing (Table 1) revealed a markedly prolonged activated partial thromboplastin time (APTT) of 48.1 s, which was fully corrected by 1:1 mixing with normal pooled plasma (35.6 s) but remained markedly prolonged after mixing with FVIII-deficient plasma (81.6 s), indicating a coagulation factor deficiency rather than an inhibitor. VWF:Ag (6.8 IU/dL), VWF:GPIbR (0.1 IU/dL), and FVIII:C (8.7 IU/dL) were all markedly reduced, indicating a severe VWD phenotype. Because these parameters alone do not define the VWD subtype, multimer analysis was further performed to assess qualitative abnormalities of VWF. The proband’s mother, who had a non-O blood group, showed normal VWF:GPIbR but a mildly reduced VWF:Ag (58.9 IU/dL), with FVIII:C and APTT within normal limits. She had no overt bleeding manifestations. The father and the proband’s sister had normal routine coagulation profiles and no overt bleeding manifestations.

### 3.2. Genetic Identification and Pedigree Analysis

**Figure 1:**
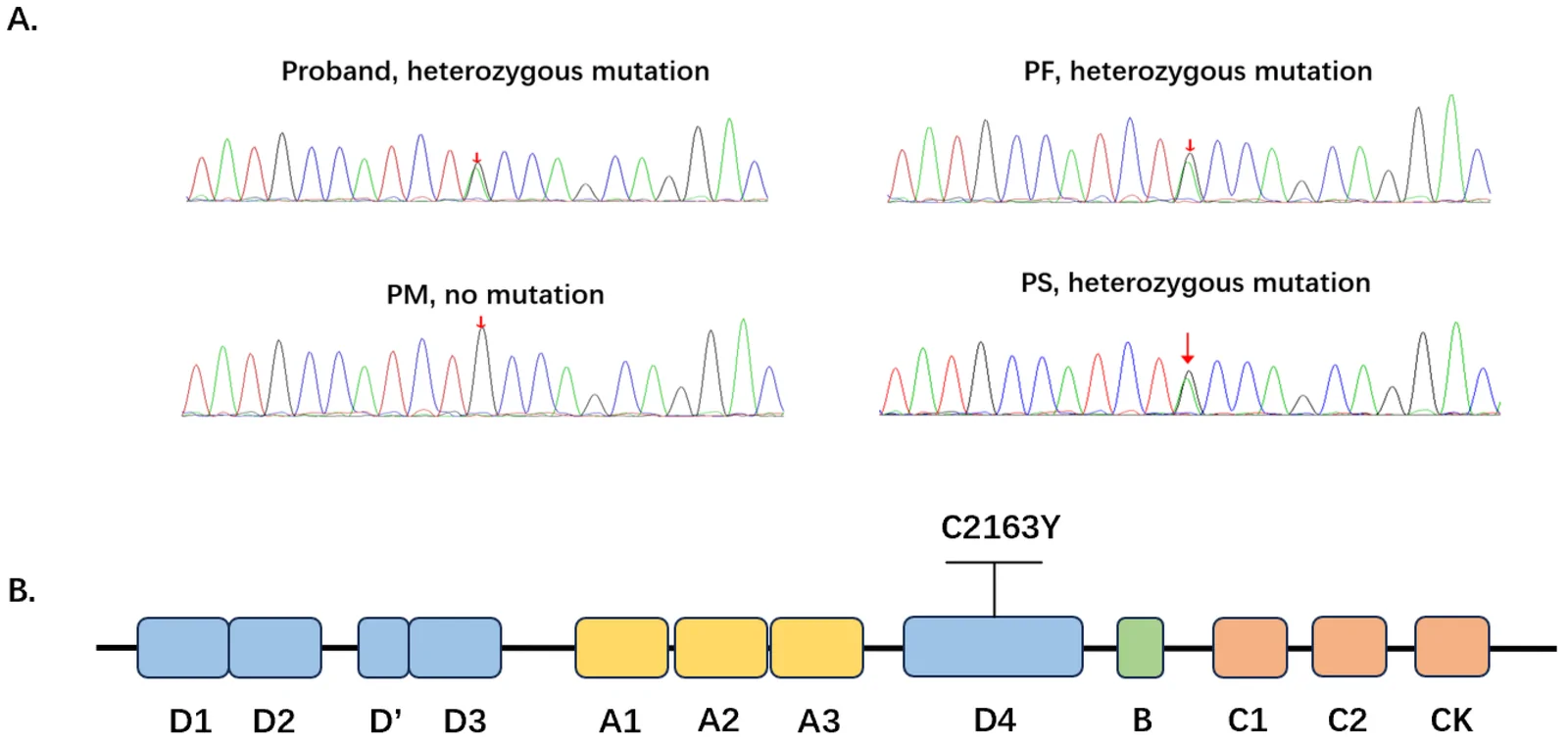
Genetic identification of the VWF c.6488G>A (p.Cys2163Tyr) variant. (**A**) Sanger sequencing chromatograms showing the heterozygous c.6488G>A (p.Cys2163Tyr) variant in the proband, father, and sister, while the mother carries the wild-type allele. The red arrows indicate the variant position, and the differently colored traces represent the four nucleotide bases: adenine (green), cytosine (blue), guanine (black), and thymine (red). (**B**) Schematic diagram of the VWF domain structure indicating the location of the p.Cys2163Tyr variant within the D4 domain. The differently colored boxes represent the distinct structural domains of VWF.

Targeted next-generation sequencing of genomic DNA from the proband identified a heterozygous c.6488G>A variant in exon 37 of the VWF gene (NM_000552), predicted to substitute cysteine for tyrosine at position 2163 of pre-pro-VWF (p.Cys2163Tyr). No additional coding or canonical splice-site pathogenic variant in VWF was identified by this short-read targeted sequencing approach. This residue is located within the D4 domain (Figure 1B). The variant was classified as a variant of uncertain significance (VUS) according to the ACMG/AMP guidelines. Although in silico tools predicted a deleterious effect, the incomplete segregation with the severe phenotype prevented classification as definitively pathogenic in this family. This variant has been recorded in ClinVar and previously reported in patients with VWD, including homozygous and compound heterozygous cases identified through the ThromboGenomics high-throughput sequencing study [20]. Therefore, p.Cys2163Tyr should not be regarded as a novel VWF variant. Although this variant has been reported previously, detailed functional characterization of its effect on VWF secretion and multimer formation has remained limited.

**Figure 2:**
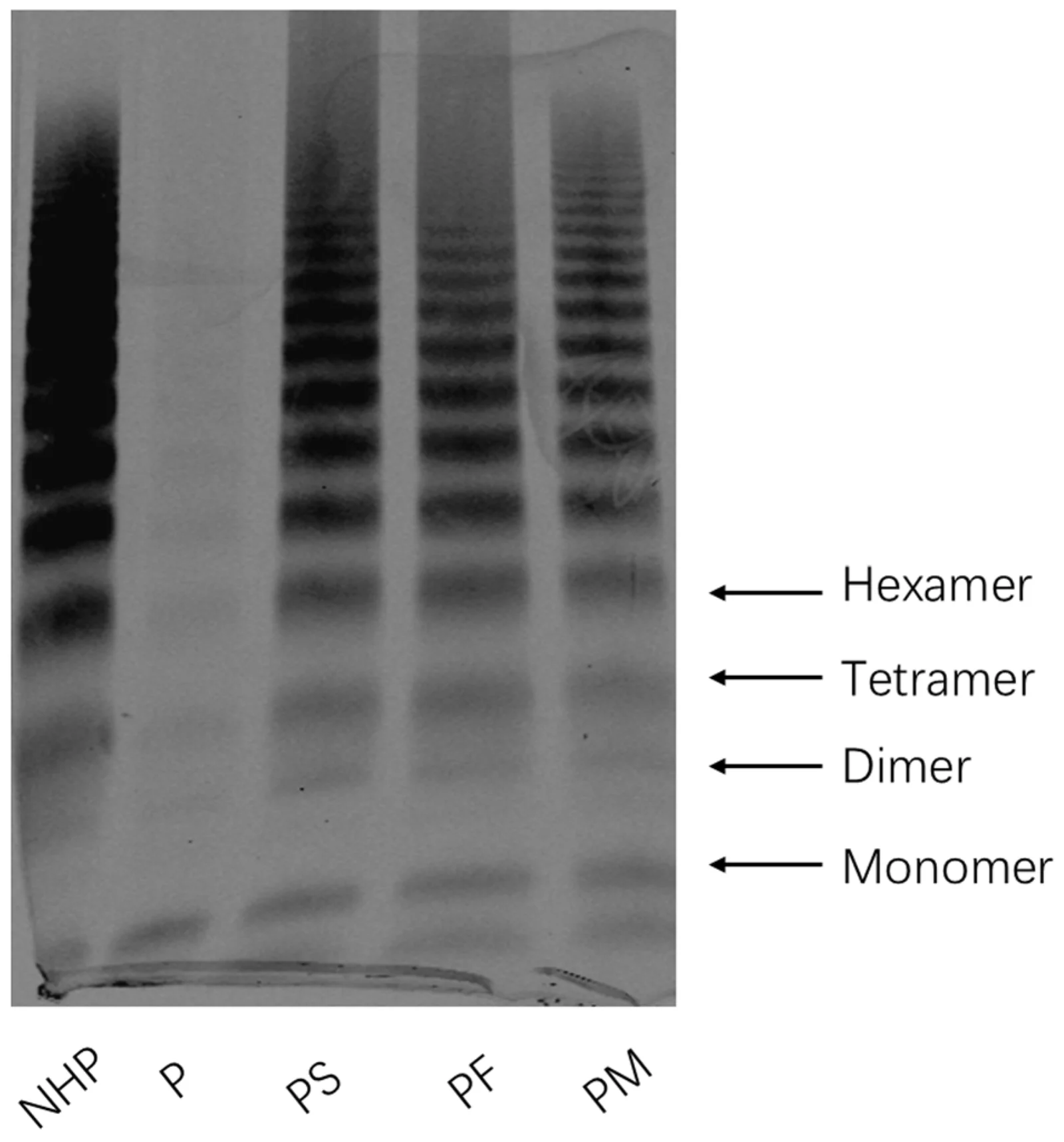
Plasma VWF multimer analysis in the study pedigree. Plasma samples from the proband and family members were analyzed by non-reducing 1.5% SDS–agarose gel electrophoresis followed by immunoblotting with an anti-VWF antibody. Normal human plasma (NHP) was used as a reference control. The proband (P) showed a markedly reduced overall VWF signal with a clear loss of high-molecular-weight (HMW) multimers, supporting classification of the phenotype as severe type 2A VWD [21,22]. The proband’s mother (PM) and sister (PS) displayed multimer distributions comparable to NHP, whereas the proband’s father (PF) exhibited a mild reduction in HMW multimers but had no overt bleeding manifestations, suggesting a subclinical effect of the variant in the heterozygous state. P, proband; PF, proband’s father; PM, proband’s mother; PS, proband’s sister; NHP, normal human plasma. Original uncropped Western blot images are provided in Supplementary Material.

Sanger sequencing in the available family members confirmed the heterozygous c.6488G>A variant in the proband, his father, and his sister, whereas the proband’s mother carried only the wild-type allele (Figure 1A). Despite carrying the variant, both the father and the sister were clinically asymptomatic, with the father showing only a mild reduction in HMW multimers (Figure 2). In contrast, the proband’s mother, who did not carry the variant, exhibited a mildly reduced VWF:Ag level (Table 1). Together, these findings indicate incomplete segregation between the heterozygous p.Cys2163Tyr variant and the severe phenotype, suggesting that p.Cys2163Tyr alone is unlikely to fully explain the proband’s disease severity. An additional unidentified VWF defect or genetic modifier, possibly inherited from the maternal side, may contribute to the severe phenotype.

### 3.3. Genotype–Phenotype Correlation of the p.Cys2163Tyr Variant

To identify the VWF gene mutation in the proband and family members, we extracted genomic DNA from the proband’s peripheral blood for high-throughput sequencing. Sequencing results revealed a G-to-A mutation at position 6488 of the VWF gene, resulting in the substitution of cysteine at position 2163 with tyrosine. Cys2163 is located within the VWF D4 domain (Figure 1A). Based on these sequencing results, we designed primers to amplify the nucleotide sequence flanking the mutation site and performed Sanger sequencing verification. Results confirmed that the proband carried a heterozygous VWF c.6488G>A (p.Cys2163Tyr) variant. The same variant was detected in the proband’s father and sister, both of whom were asymptomatic or exhibited only mild laboratory abnormalities. Notably, the proband’s mother did not carry the p.Cys2163Tyr variant but showed mildly reduced VWF levels. These findings suggest that the p.Cys2163Tyr variant alone is insufficient to produce a severe phenotype (Figure 1B).

### 3.4. The P.cys2163tyr Variant Markedly Reduces VWF Secretion in Hek293t Cells

**Figure 3:**
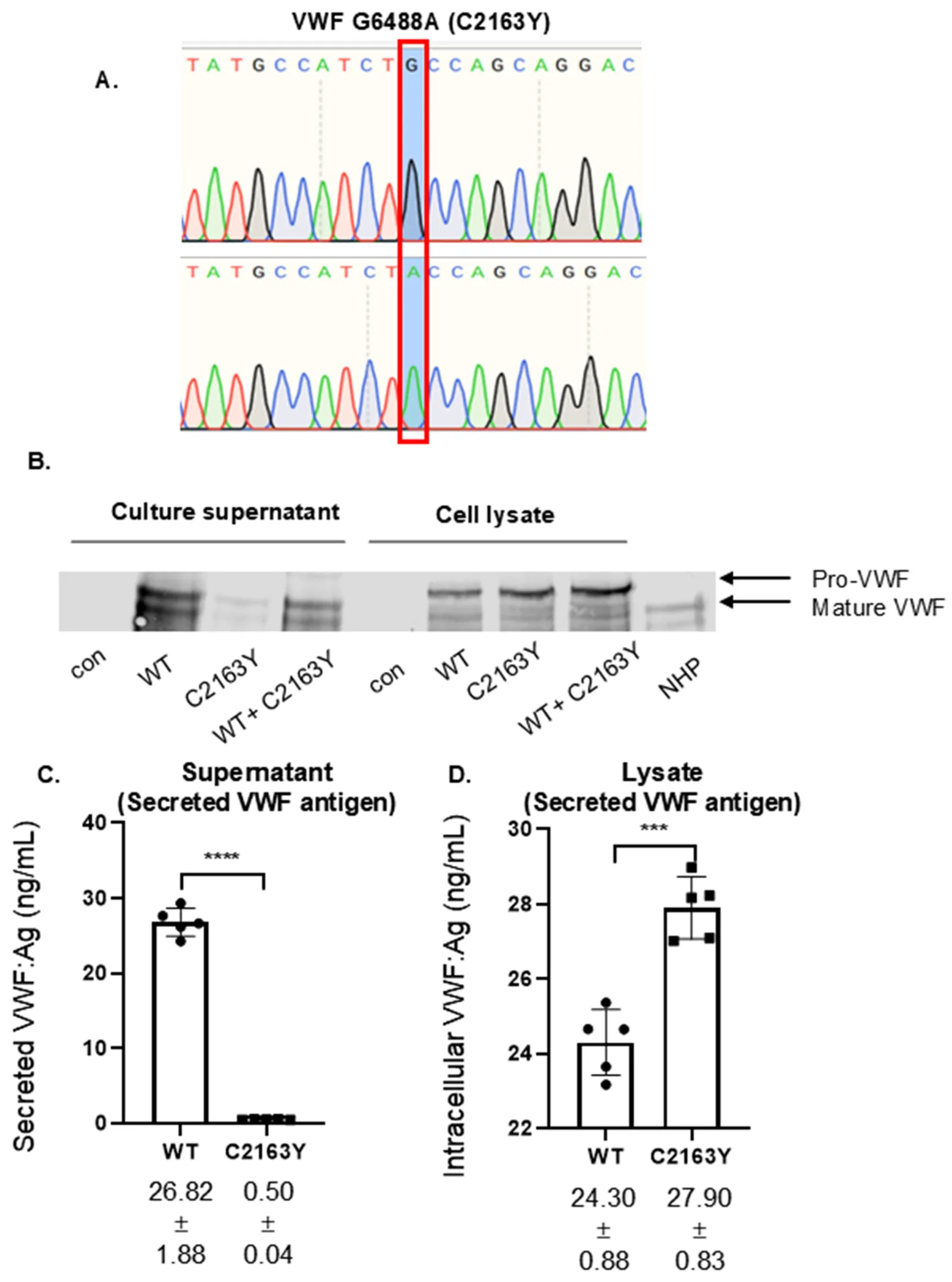
Expression and secretion of wild-type and p.Cys2163Tyr VWF in HEK293T cells. (**A**) Sequence confirmation of the c.6488G>A substitution introduced by site-directed mutagenesis. The red box highlights the c.6488G>A substitution site. (**B**) Western blot analysis of VWF in cell lysates and concentrated culture supernatants collected 72 h after transfection with empty vector, WT, p.Cys2163Tyr, or WT + p.Cys2163Tyr (1:1) constructs. Samples were analyzed under reducing conditions and immunoblotted with an anti-VWF antibody. Compared with WT, the p.Cys2163Tyr variant showed markedly reduced levels in the culture supernatant, whereas the WT + p.Cys2163Tyr co-expression group showed an intermediate secretion pattern. Intracellular VWF remained detectable in all transfected groups. (**C**) ELISA-based quantification of VWF antigen in conditioned media showed a marked reduction in secreted recombinant VWF in the p.Cys2163Tyr group compared with WT. (**D**) ELISA-based quantification of intracellular VWF antigen in cell lysates showed that intracellular mutant VWF was preserved and modestly higher than WT. Data are presented as mean ± SD from independent experiments (*n* = 5). Statistical comparisons between WT and p.Cys2163Tyr were performed using an unpaired two-tailed Student’s *t*-test. For secreted VWF, *p* < 0.0001; for intracellular VWF, *p* = 0.0002. **** *p* < 0.0001. *** *p* < 0.001.

To examine the effect of the p.Cys2163Tyr variant on VWF expression and secretion, the variant was introduced into the wild-type pSVHVWF expression construct by site-directed mutagenesis, with successful introduction confirmed by Sanger sequencing (Figure 3A). HEK293T cells were transfected with empty vector, wild-type VWF (WT), VWF-p.Cys2163Tyr, or both WT and VWF-p.Cys2163Tyr constructs at a 1:1 ratio, and conditioned media and cell lysates were collected 72 h post-transfection.

Western blot analysis under reducing conditions revealed that VWF was readily detected in the conditioned medium of WT-transfected cells, whereas it was markedly reduced in the medium of VWF-p.Cys2163Tyr–transfected cells (Figure 3B). In contrast, intracellular VWF levels in the corresponding cell lysates were comparable between the WT and VWF-p.Cys2163Tyr groups. The WT + VWF-p.Cys2163Tyr co-expression group displayed an intermediate secretion pattern, with more secreted VWF than the VWF-p.Cys2163Tyr group but less than the WT group. ELISA-based quantification confirmed these observations. Secreted VWF in conditioned media was markedly reduced in the VWF-p.Cys2163Tyr group compared with the WT group (0.50 ± 0.04 ng/mL vs. 26.82 ± 1.88 ng/mL, mean ± SD, *n* = 5 independent transfections; Student *t*-test, *p* < 0.0001; Figure 3C). In contrast, intracellular VWF antigen in cell lysates was preserved in the VWF-p.Cys2163Tyr group and was slightly higher than in the WT group (27.90 ± 0.83 ng/mL vs. 24.30 ± 0.88 ng/mL, mean ± SD, *n* = 5; Student *t*-test, *p* = 0.0002; Figure 3D). Together, these findings indicate that the p.Cys2163Tyr variant does not abolish intracellular VWF production but markedly reduces the amount of VWF released into the culture medium, consistent with a secretion-defective phenotype in vitro.

### 3.5. Multimer Analysis of Recombinant VWF Cys2163tyr Protein in Hek293t Cells

To examine the effect of the p.Cys2163Tyr variant on dimer and multimer assembly, recombinant VWF in cell lysates and conditioned media from transfected HEK293T cells was analyzed by SDS–agarose gel electrophoresis under non-reducing conditions. VWF-p.Cys2724Tyr, a dimerization-defective mutant previously characterized by our group [7], was included as a control.

**Figure 4:**
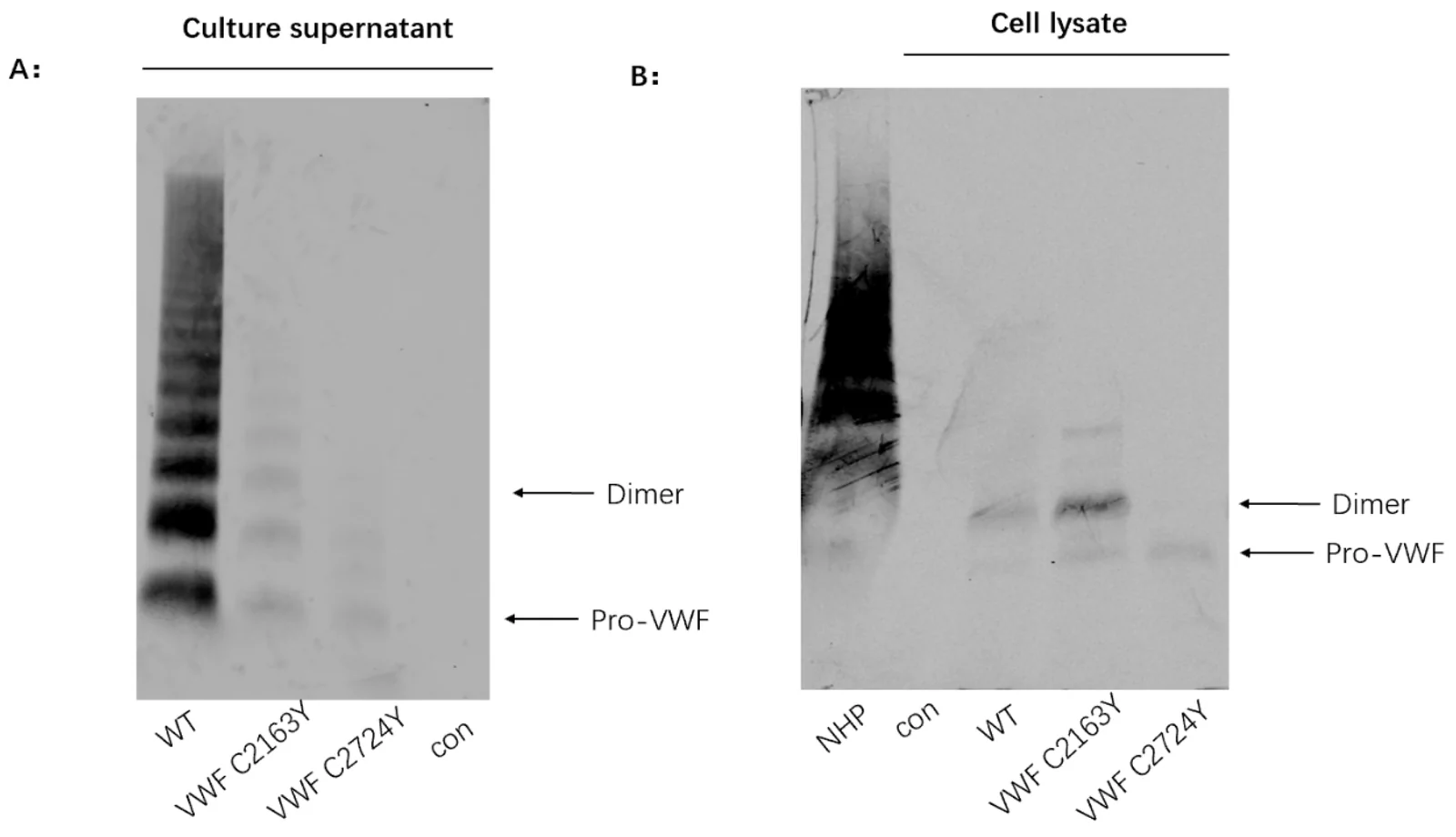
Reduced multimer formation of p.Cys2163Tyr in vitro. Multimer analysis of VWF in cell lysates (**A**) and culture supernatants (**B**) from HEK293T cells transfected with empty vector, wild-type VWF, p.Cys2163Tyr VWF, or the dimerization-defective control mutant p.Cys2724Tyr VWF. Proteins were separated by SDS–agarose gel electrophoresis under non-reducing conditions and detected by immunoblotting. Wild-type VWF formed normal high-molecular-weight multimers in the supernatant, whereas the p.Cys2163Tyr variant showed a marked reduction in high-molecular-weight multimers, with only trace amounts of intermediate-molecular-weight species detected. In cell lysates, the p.Cys2163Tyr variant was detectable but exhibited altered multimer patterns compared with wild-type VWF.

In cell lysates, both WT VWF and VWF-p.Cys2163Tyr formed dimers together with some higher-order multimers, whereas VWF-p.Cys2724Tyr remained in its precursor (monomeric) form and failed to dimerize, as expected (Figure 4A). In conditioned media, WT VWF displayed a full range of multimers including normal high-molecular-weight (HMW) species, whereas VWF-p.Cys2163Tyr showed a marked reduction in HMW multimers, with predominantly low-molecular-weight species and only trace amounts of intermediate-molecular-weight multimers detected (Figure 4B). These findings indicate that the p.Cys2163Tyr variant does not impair dimer formation, but disrupts the subsequent assembly and/or secretion of high-molecular-weight multimers.

## 4. Discussion

In this study, we functionally characterized the VWF c.6488G>A (p.Cys2163Tyr) variant, located within the D4 domain, in a patient with a severe VWD phenotype showing loss of high-molecular-weight multimers. In vitro, the variant was associated with markedly reduced extracellular VWF and impaired high-molecular-weight multimer formation, while intracellular VWF antigen was preserved. These findings suggest that the variant affects post-translational maturation, trafficking, secretion, and/or multimer accumulation rather than VWF synthesis alone.

The variant p.Cys2163Tyr has been previously reported in VWF databases (e.g., ClinVar Miner) and in the literature [23]; however, functional evidence explaining how this substitution affects VWF biosynthesis and multimer formation has been limited. The pedigree analysis indicates incomplete segregation between the heterozygous p.Cys2163Tyr variant and the severe phenotype, suggesting that this variant alone is unlikely to fully account for the proband’s disease severity. The proband inherited the variant from his father, and both the father and the sister carry the same variant without overt bleeding manifestations, indicating that heterozygosity for p.Cys2163Tyr is not sufficient by itself to cause a severe phenotype in this family. However, the father showed a mild reduction in HMW multimers, suggesting that the variant may exert a subclinical effect on VWF processing even in the heterozygous state, although this effect was insufficient to produce clinically apparent bleeding. In contrast, the proband’s mother, who did not carry the p.Cys2163Tyr variant, exhibited a mildly reduced VWF:Ag despite her non-O blood group, which would normally be associated with higher VWF levels. This finding raises the possibility of an additional undetected VWF defect, regulatory variant, or genetic modifier affecting the maternal contribution to the proband’s phenotype. Although no second pathogenic variant was identified by targeted next-generation sequencing, we did not perform MLPA, long-read sequencing, CNV analysis, or RNA studies. Therefore, large deletions or duplications, complex structural variants, deep intronic variants, regulatory defects, or transcript-level abnormalities cannot be excluded. Taken together, these observations support a model in which combined defects affecting both VWF alleles underlie the severe phenotype of the proband, although further genetic investigation is required to confirm this hypothesis.

Given that Cys2163 is located in the D4 domain and that one of the major func-tions of this domain is ADAMTS-13 binding, we speculate that Cys2163Tyr may en-hance the interaction between VWF and ADAMTS-13, thereby promoting proteolysis of VWF multimers. This may explain why small amounts of low- and intermediate-molecular-weight multimers were detected in the supernatant of 293T cell cultures but were nearly absent in the proband’s plasma, as ADAMTS-13 is present in plasma but not in the 293T cell culture supernatant. This mechanism may also account for the mild loss of high-molecular-weight VWF multimers observed in the plasma of the proband’s father and sister. Similarly, it may explain the markedly reduced VWF:GPIbR/VWF:Ag ratio and supports our diagnosis of type 2A VWD.

In our in vitro expression experiments, the p.Cys2163Tyr variant was associated with markedly reduced secretion and severely impaired multimer formation in the secreted fraction, while VWF dimers remained detectable in cell lysates and intracellular VWF antigen was preserved by ELISA. These findings indicate that the variant does not abolish dimerization or intracellular protein production, but instead disrupts later stages of VWF biosynthetic maturation. Several non-mutually exclusive mechanisms may explain the intracellular accumulation and reduced extracellular VWF, including endoplasmic reticulum retention due to misfolding, increased intracellular degradation, abnormal ER-to-Golgi trafficking, and impaired multimer assembly. In this context, the observed multimer abnormalities are more likely secondary to defective intracellular processing and trafficking than to a purely isolated defect in multimer assembly. This interpretation is consistent with the established biosynthetic pathway of VWF, in which dimerization occurs in the endoplasmic reticulum, whereas further multimer assembly and post-translational processing occur during Golgi trafficking before secretion [1,24]. In addition, the WT + p.Cys2163Tyr co-expression group showed an intermediate secretion pattern on immunoblotting, suggesting that under a heterozygous-like condition the mutant is associated with partial reduction in secreted VWF, but without evidence of a complete blockade of WT secretion.

Our findings support a model in which substitution of this conserved cysteine residue in the D4 domain impairs the post-translational maturation of VWF, thereby reducing the amount of properly processed and secreted protein. The reduced proportion of HMW multimers in the secreted fraction may reflect a direct impairment of multimer assembly within the Golgi apparatus, a secondary consequence of defective secretion favoring release of lower-order multimers, or a combination of both mechanisms. Further studies examining the intracellular multimer composition prior to secretion would help clarify the relative contribution of each process, and represent a valuable direction for future investigation.

Previous studies on the VWF D4 domain have mainly focused on its plasma functions, including binding to ADAMTS13 [25,26], and regulation of the conformational response of VWF to shear stress [27,28]. In contrast, our findings extend the functional scope of the D4 domain by supporting its involvement in maintaining VWF structural stability, which is important for VWF secretion and multimerization. This represents an important addition to our understanding of D4 domain function.

A pathogenic role for the D4 domain in VWF biosynthesis has been suggested by earlier observations. Dubois et al. reported two frameshift variants, p.(Pro2145Thrfs*5) and p.(Cys2216Phefs*9), that impair VWF biosynthesis and secretion in vitro [12]; however, because these variants introduce premature termination codons, the resulting defects likely reflect the loss of downstream sequences rather than dysfunction confined to the D4 domain. Importantly, in the same study, Cys2139 and Cys2163 were predicted to form a disulfide bridge within the D4 domain [12], implying that substitution of Cys2163 would disrupt this bond and potentially compromise D4 folding [12]. More recently, Mobayen et al. systematically characterized nine missense variants within the D4 and C-terminal domains of VWF, demonstrating that several of them, including amino acid substitutions in the D4 domain, are sufficient to impair VWF secretion in HEK293T cells [29]. Our characterization of the p.Cys2163Tyr variant extends these observations by providing functional evidence that disruption of a specific D4-domain cysteine residue—likely through loss of a structural disulfide bond—severely impairs VWF secretion and HMW multimer assembly.

In normal plasma, VWF circulates as a heterogeneous population of multimers ranging from dimers to ultra-large forms, and multimer size is a key determinant of its functional activity [1,4]. The marked loss of HMW multimers observed in the proband provides a plausible explanation for the severe bleeding phenotype; however, because the same heterozygous variant was present in asymptomatic relatives, the clinical severity is unlikely to be explained by p.Cys2163Tyr alone.

Study limitations. Our study has several limitations. First, the possibility of an additional genetic defect contributing to the proband’s severe phenotype remains unresolved. MLPA, dedicated CNV analysis, long-read sequencing, and RNA studies were not performed; therefore, exon-level deletions or duplications, complex structural variants, deep intronic variants, regulatory variants, and transcript abnormalities cannot be excluded [30]. Second, the functional studies were performed in HEK293T cells, which are useful for recombinant VWF expression but lack endothelial-specific Weibel–Palade body formation and regulated secretion. Therefore, the findings should be validated in endothelial-relevant models in future studies. Third, although Western blotting and ELISA demonstrated preserved or increased intracellular mutant VWF with markedly reduced extracellular VWF, immunofluorescence colocalization, pulse-chase experiments, and ER/Golgi trafficking analyses were not performed. Thus, the relative contributions of ER retention, intracellular degradation, abnormal trafficking, and impaired multimer maturation remain to be defined. Finally, no structural or biochemical analyses were performed to directly verify whether the p.Cys2163Tyr substitution disrupts the predicted Cys2139–Cys2163 disulfide bond or alters D4 domain folding, and confirming this mechanism will require future studies using purified recombinant proteins or structural approaches. This proposed mechanism therefore remains inferential.

## 5. Conclusions

In conclusion, our findings support that VWF Cys2163Tyr is a functionally deleterious variant in vitro, characterized by markedly impaired secretion and reduced high-molecular-weight multimer formation. In vitro, the variant retained normal intracellular expression and dimer formation but showed markedly reduced extracellular secretion and impaired HMW multimer accumulation. These findings provide functional evidence that the D4 domain contributes to VWF biosynthesis, secretion, and multimer homeostasis, in addition to its previously described roles in ADAMTS13 binding and shear-stress-dependent conformational regulation. Pedigree analysis further suggests that the severe phenotype of the proband is unlikely to be explained by the heterozygous p.Cys2163Tyr variant alone and may involve an additional unidentified genetic defect or modifier. Together, these findings expand the functional understanding of the VWF D4 domain and highlight the value of integrating clinical, genetic, and functional analyses when interpreting VWF variants with incomplete genotype–phenotype correlation.

## Figures and Tables

**Table 1:** Coagulation parameters in the proband and family members.

Blood Test	Proband (P)	Mother (PM)	Father (PF)	Sister (PS)
VWF:GPIbR (IU/dL)	0.10	53.40	44.60	63.90
VWF:Ag (IU/dL)	6.80	58.90	49.10	64.10
APTT (normal control) (s)	32.0	32.0	32.0	32.0
APTT (before mixing) (s)	48.1	32.0	34.1	34.8
APTT (mixing with normal plasma) (s)	35.6	32.1	33.4	32.7
APTT (mixing with FVIII-deficient plasma) (s)	81.6	57.0	59.5	56.7
FVIII:C (IU/dL)	8.70	75	57.50	77.60

VWF:Ag, VWF:GPIbR, and FVIII:C were markedly reduced in the proband, consistent with severe VWD. The mother showed isolated mild VWF:Ag reduction without the Cys2163Tyr mutation. APTT mixing studies demonstrated complete correction with normal plasma but not with FVIII-deficient plasma, confirming concomitant FVIII deficiency. The interpretation of VWF levels was based on ABO-specific reference ranges, as ABO blood group is a major determinant of circulating VWF and FVIII levels [17,18,19]. Reference ranges for VWF activity (VWF:GPIbR) vary by ABO blood group: 40.3–125.9 IU/dL for blood group O, and 48.8–163.4 IU/dL for non-O blood groups (A, B, and AB). Reference ranges for VWF antigen (VWF:Ag) vary by ABO blood group: 42.0–140.8 IU/dL for blood group O, and 66.1–176.3 IU/dL for non-O blood groups. Reference range for APTT: 25.4–38.4 s. Reference range for FVIII:C: 50–150 IU/dL.

## Data Availability

The original contributions presented in this study are included in the article/Supplementary Material. Further inquiries can be directed to the corresponding author(s).

## Supplementary Materials

The following supporting information can be downloaded at: https://www.mdpi.com/article/10.3390/biom16081088/s1, Original uncropped Western blot images are provided in Supplementary Material.
